# Machine learning of flow cytometry data reveals the delayed innate immune responses correlate with the severity of COVID-19

**DOI:** 10.3389/fimmu.2023.974343

**Published:** 2023-01-26

**Authors:** Jing Zhu, Tunan Chen, Xueying Mao, Yitian Fang, Heqi Sun, Dong-Qing Wei, Guangfu Ji

**Affiliations:** ^1^ National Key Laboratory for Shock Wave and Detonation Physics Research, Institute of Fluid Physics, Chinese Academy of Engineering Physics, Mianyang, China; ^2^ Department of Neurosurgery and Key Laboratory of Neurotrauma, Southwest Hospital, Third Military Medical University (Army Medical University), Chongqin, China; ^3^ State Key Laboratory of Microbial Metabolism, Shanghai-Islamabad-Belgrade Joint Innovation Center on Antibacterial Resistances, Joint Laboratory of International Cooperation in Metabolic and Developmental, Sciences, Ministry of Education and School of Life Sciences and Biotechnology, Shanghai Jiao Tong University, Shanghai, China; ^4^ Peng Cheng Laboratory, Shenzhen, China

**Keywords:** delayed innate immune responses, relevance scores, COVID-19, neural network, dynamics model

## Abstract

**Introduction:**

The COVID-19 pandemic has posed a major burden on healthcare and economic systems across the globe for over 3 years. Even though vaccines are available, the pathogenesis is still unclear. Multiple studies have indicated heterogeneity of immune responses to SARS-CoV-2, and potentially distinct patient immune types that might be related to disease features. However, those conclusions are mainly inferred by comparing the differences of pathological features between moderate and severe patients, some immunological features may be subjectively overlooked.

**Methods:**

In this study, the relevance scores(RS), reflecting which features play a more critical role in the decision-making process, between immunological features and the COVID-19 severity are objectively calculated through neural network, where the input features include the immune cell counts and the activation marker concentrations of particular cell, and these quantified characteristic data are robustly generated by processing flow cytometry data sets containing the peripheral blood information of COVID-19 patients through PhenoGraph algorithm.

**Results:**

Specifically, the RS between immune cell counts and COVID-19 severity with time indicated that the innate immune responses in severe patients are delayed at the early stage, and the continuous decrease of classical monocytes in peripherial blood is significantly associated with the severity of disease. The RS between activation marker concentrations and COVID-19 severity suggested that the down-regulation of IFN-γ in classical monocytes, Treg, CD8 T cells, and the not down-regulation of IL_17a in classical monocytes, Tregs are highly correlated with the occurrence of severe disease. Finally, a concise dynamic model of immune responses in COVID-19 patients was generalized.

**Discussion:**

These results suggest that the delayed innate immune responses in the early stage, and the abnormal expression of IL-17a and IFN-γ in classical monocytes, Tregs, and CD8 T cells are primarily responsible for the severity of COVID-19.

## 1 Introduction

Coronavirus disease 2019 (COVID-19), caused by the novel human pathogen severe acute respiratory syndrome coronavirus 2(SARS-CoV-2), is a serious disease that has resulted in widespread global morbidity and mortality. Clinical manifestations of COVID-19 are heterogeneous, with about 80% people experiencing asymptomatic or moderate symptom, the other patients develop severe symptom which may progress to acute respiratory distress syndrome (ARDS) (1, 2). Even though vaccines are available, the pathogenesis of COVID-19 is still unclear. To optimally manage the pandemic, there is an urgent need to understand the host immune responses in COVID-19 patients.

High-throughput single-cell technologies such as flow cytometry and mass cytometry, which can measure features on millions of individual cells, are well suited to support studies of the heterogeneity of immune responses and of how immune cells interact with other host cells and pathogens. Identification of host immunological correlated factors for disease severity is one of the most common application of single-cell technologies (3). Innate immune cells like basophils (4), monocytes (5), plasma DCs (4, 6), and NK cells (6) were reported with reduced abundances in peripheral blood of COVID-19 patients, and with greater reductions in individuals with severe disease than those with moderate disease. While other innate immune cells like neutrophils (7, 8), eosinophils (9) have been shown increased abundances in COVID-19 patients, especially severe patients. And the neutrophil-to-lymphocyte ratio (10, 11) was also reported to be associated with severity of illness. What’s more, the numbers of SARS-CoV-2 specific B cells were also found increased from 1-3 months (12) after symptom onset, but with abnormal expansion of antibody-secreting cells in severe patients rather than moderate patients (13), which raised the question about the role of B cell responses in COVID-19 patients. Nevertheless, T cell responses in COVID-19 patients are more controversially, there were evidences of terminally differentiated T cells in severe disease (10, 14), other study (15) suggested that CD8 T cells in severe patients might in a hyperactive state by expressing high level of nature killer cell related markers and increased cytotoxicity. It is not clear whether the T cells in severe patients are exhausted or just highly activated. Multiple studies (14, 16–18) have indicated heterogeneity of immune responses to SARS-CoV-2, and potentially distinct patient immune types that might be related to disease features.

However, those immunological correlated factors for disease severity in previous studies were mainly inferred by comparing the differences of cell counts or bio-marker expression levels between moderate and sever patients, some immunological features may be subjectively overlooked. In this study, the relevance scores(RS) (19–26) between immunological features and the severity of COVID-19 are objectively calculated through neural network, this calculation method belongs to the feature importance explainability approaches(explanation for AI system’s decisions) (25), these values reflect which features played a more critical role in the decision-making process. To the best of our knowledge, this is the first time that COVID-19 patients’ cytometry data are analyzed by the explainability approach of AI system. Firstly, we collected two publicly available flow cytometry data sets containing peripheral blood information of COVID-19 patients from the Flow Repository website (27). Secondly, we used the PhenoGraph algorithm (28) to robustly cluster these patients’ cells into phenotypically distinct subpopulations. Thirdly, we constructed a neural network with these immune cell counts or activation marker concentrations of particular immune cells as the input neurons, the disease severity as the output neuron. Fourthly, we calculated the RS value between input neurons and output neuron through the Layer-wise Relevance Propagation(LRP) algorithm (20), then we compared these RS between immune cell counts and disease severity at different stages, and analyzed the RS between activation marker concentrations and disease severity on particular immune cells. Finally, we generalized a concise dynamic model of immune responses in COVID-19 patients. These results suggested that the delayed innate immune responses in the early stage is primarily responsible for the severity of COVID-19.

## 2 Methods

### 2.1 Acquisition of data sets

To understand the host immune responses to SARS-CoV-2 infection, the publicly available individual flow cytometry data sets were selected from the Flow Repository (http://flowrepository.org/) (27) under accession number FR-FCM-Z36F (29) and FR-FCM-Z2KP (30). Detailed information of samples in these data sets can be found in the original research papers and on the Flow Repository website. A total of 145 samples were obtained from these data sets and a summary of these data sets can be found in **Table 1**. Relevance data sets were identified from the query “COVID-19”. Selection was primarily focus on the integrity of severity categories: health control, mild/moderate and severe, the specific of patient’s illness time, the uniformity of the patient’s condition distribution, and the staining strategy(which could identify the lymphocyte subsets). Due to the differenct staining strategies of the data sets in Flow Repository, it is infeasible to merge them into a large and consistent data set. Finally, one mass cytometry and one flow cytometry data set were selected from 22 COVID-19 related data sets. The data set FR-FCM-Z36F was collected from a cohort of hospitalized COVID-19 patients and healthy controls to identify dynamic disease-associated changes in circulating immune cell frequency and phenotype, it will be used for calculating the RS between immune cell counts and the severity of COVID-19 in time. The data set FR-FCM-Z2KP was collected to analysis the activation markers produced by PBMC from COVID-19 patients, it will be used for calculating the RS between activation marker concentrations produced by PBMC and the severity of COVID-19.

**Table 1:** Publicly available data sets from the Flow repository database included in analysis.

Repository ID	FCS File Numbers	Study Design	Sample Source	Sample Description	Detecting Instrument	The RS calculated by neural network
FR-FCM-Z36F (29)	96	Apply a streamlined CYTOF workflow to characterize whole blood samples to identify dynamic disease-associated changes in circulating immune cell frequency and phenotype.	Human whole blood	Severe: 36 (Early stage: 11 Middle stage: 10 Late stage: 15) Moderate: 48 (Early stage: 28 Middle stage: 13 Late stage: 7) Healthy: 12	Mass cytometry	The RS between immune cell counts and COVID-19 severity.
FR-FCM-Z2KP (30)	49	Analysis of cytokine secretion in PBMC of patients with COVID-19.	PBMC	Severe: 17 Severe_to_Moderate: 6 Moderate: 20 Healthy: 6	BD Symphony	The RS between activation marker concentrations of immune cells and COVID-19 severity.

### 2.2 Data pre-processing (Clustering by PhenoGraph algorithm)

The PhenoGraph algorithm (28) was used for robustly clustering the peripheral blood cells of COVID-19 patients into phenotypically distinct subpopulations. The algorithm was run on the R-based (31) application. Samples(in.fcs files) were first pre-processed: margin events were filtered out, live single cells were gated (11). Then these cleaned data were used for PhenoGraph training. To address patient specific variability and to understand immune cells dynamics shared between samples, PhenoGraph clusters were merged, and data were transformed with an arcsin*h* transformation with cofactor 5. The k-nearest neighbor was set to be 30 for data set Z36F, and 100 for data set Z2KP.

After the PhenoGraph clustering, the percentage of peripheral blood cells(cell counts) for each cluster (phenotypically distinct subpopulations) of patients are recorded(see in **S1 File**), these data are tensors of floating point numbers distributed between 0 and 1, which can be directly used as the input of neural network. And the expression matrix of each cluster(activation marker concentration of cells) are recorded also(see in **S2 File**), all data were compressed with an arcsin*h* transformation with cofactor 5, the missing value of patients is set to 0, these data will be used as the input of neural network as well.

### 2.3 Relevance scores calculated by neural network

Given a trained neural network that models a scalar-valued prediction score for each target output, and given an input vector, we are interested in computing for a RS quantifying the relevance of input vector to a considered target of interest (25). In other words, we want to analyze which features of input vector are important for the neural network’s decision toward the target.

The RS can be computed by the Layer-wise Relevance Propagation(LRP) algorithm proposed by Bach et al (19), these derivations go from upper-layer neurons to lower-layer neurons. Let *z_j_* be an upper-layer neuron, whose value in the forward pass is computed as *z_j_* =Σ*_i_ z_i_*·*w_ij_* + *b_j_* where *z_i_* is one neuron of the lower-layer, and *w_ij_*, *b_j_* are the connection weight and biases. The relevance redistribution onto lower-layer neurons *z_i_* is performed in two steps:

Step one, computing relevance messages *R*_*i*←*j*_ going from upper-layer neuron *z_j_* to lower-layer neuron *z_i_*.


(1) $$R_{i\leftarrow j}=\frac{z_{i}\;\cdot \;w_{\mathrm{ij}}\;+\frac{\epsilon \;\cdot \;sign(z_{j})\;+\;\delta \;\cdot \;b_{i}}{N}}{z_{j}\;+\;\epsilon \;\cdot \;sign(z_{j})\;}\cdot R_{j}$$

(20, 21)

where *N* is the total number of lower-layer neurons to which *z_j_* is connected, *ϵ* is a small positive number which serves as a stabilizer, and *sign(z_j_)=(lz_j≥0_-lz_j<0_)* is defined as the sign of *z_j_*. Moreover, δ is a multiplicative factor that is either set to 1.0, in which case the total relevance of all neurons in the same layer is conserved, or else it is set to 0.0, which implies that a part of total relevance is “absorbed” by the biases and that the relevance propagation rule is approximately conservative.

Since ϵ is a small stabilizer, formula (1) actually equals to


(2) $$R_{i\leftarrow j}=\left(\frac{z_{i\;}\;\cdot \;w_{i\;j}}{z_{j}}+\frac{\delta \;\cdot \;b_{j}}{N\;\cdot \;z_{j}}\right)\cdot R_{j}$$

In our experiment, we set δ=0 to ignore the effect of b_j_ to RS. Formula (2) finally reduced to


(3) $$R_{i\leftarrow j}≃\frac{z_{i}\cdot w_{ij}}{z_{j}}\cdot R_{j}$$

(19, 22)

Step two, computing relevance R_i_ going from all the neurons in upper-layer to lower-layer neuron z_i_.


(4) $$R_{i}={\sum_{j}}^{}R_{i\leftarrow j}$$

(20, 21)

**Figure 1:**
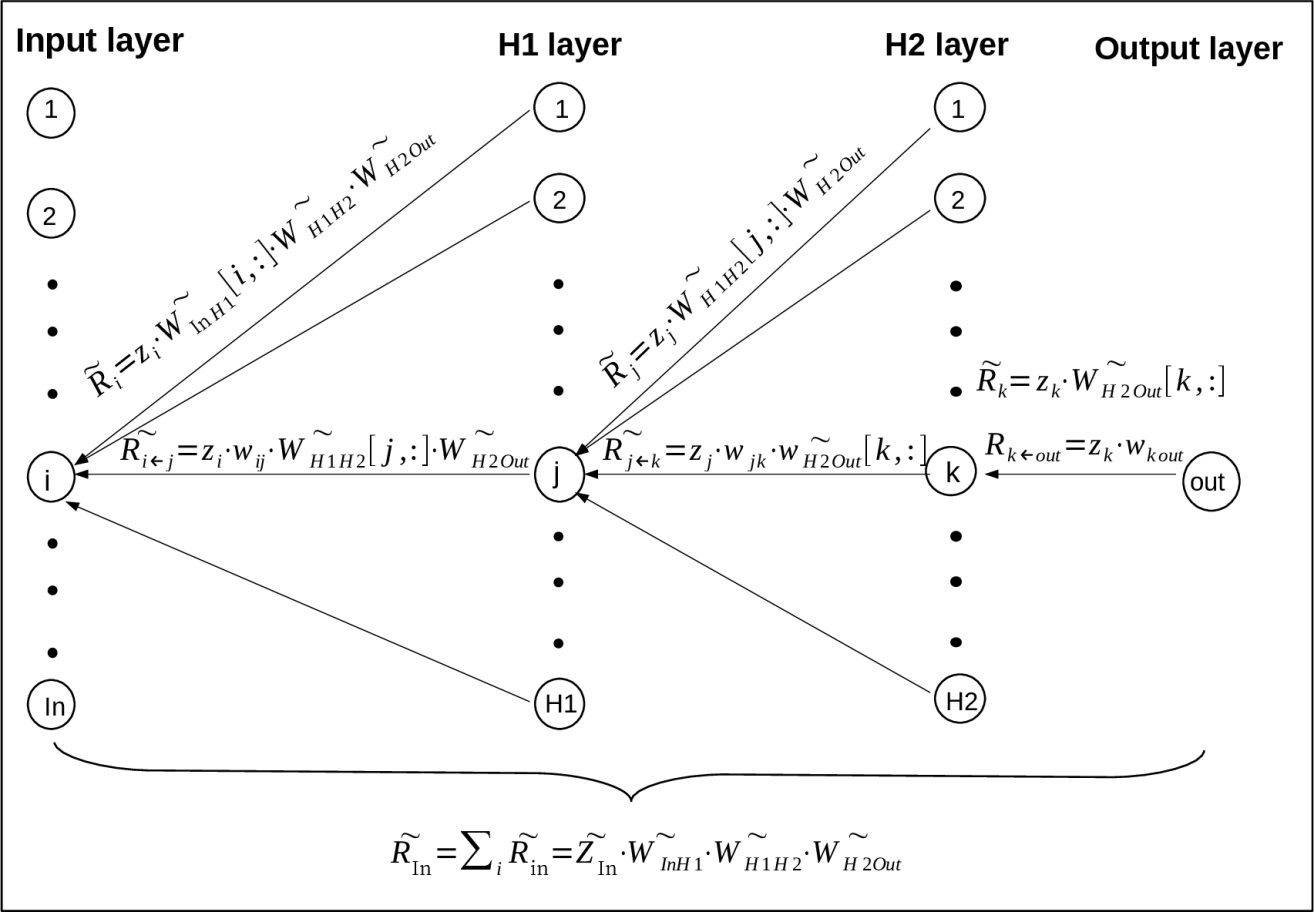
The derivation of RS value goes from upper-layer neurons to lower-layer neurons (23) based on LRP algorithm. Where the neural network has two hidden layers H1 and H2, and the output layer has only one neuron. Specifically, ‘i’, ‘j’, ‘k’ and ‘out’ represent one neuron of the input, H1, H2 and output layer, and the neuron number of these layers are ‘In’, ‘H1’, ‘H2’ and ‘Out’ respectively. *R*_*k*←*out*_ means the relevance going from the neuron *out* in output layer to neuron k in H2 layer, $\tilde{R_{k}}$ means the relevance vector going from all the neurons in output layer to neuron k in H2 layer, and so on for the other vectors.


Taking a regression neural network with two hidden layers as an example, the structure of this neural network is [In, H1, H2, Out](**Figure 1**). To calculate *R*_*k*←*out*_ going from neuron ‘*out’* in output layer to neuron ‘*k*’ in H2 layer. Since there is only one single output neuron *z_out,_* its relevance R_out_ is set to its value z_out_ (20). Plug them into formula (3):

(5) $$R_{k\leftarrow out}=z_{k}\;\cdot \;w_{k\;out}$$

where z_k_ means neuron ‘*k*’ in H2 layer, *w_k ou_*_t_ means the connection weight between neuron *‘k’* and neuron ‘*out’*.

To calculate the vector $\tilde{R_{k}}$ going from all the neurons in output layer(the actual number of neurons in output layer is ignored here to unify the representation) to neuron ‘*k*’ in H2 layer:


(6) $$\tilde{R_{k}}={\sum_{\text{out}}}^{}R_{k\leftarrow out}=z_{k}\cdot \tilde{W_{H2\;\mathrm{Out}}}\left[k,:\right]$$

where $\tilde{W_{H2\;\mathrm{Out}}}\left[k,:\right]$ means the connection vector between neuron k and all the neurons in output layer, its value is obtained by taking the *k*th row of vector $\tilde{W_{H2\;\mathrm{Out}}}$ .

$\tilde{R_{j}}$ and $\tilde{R_{i}}$ are obtained by repeating formula (5) and (6) as shown in **Figure 1**. $\tilde{R_{\text{In}}}={\sum_{i}}^{}\tilde{R_{i}}$ , which represents the relevance score going from all the neurons in H1 layer to all the neurons in input layer, is finally expressed as


(7) $$\tilde{R_{\text{In}}}={\sum_{i}}^{}\tilde{R_{i}}=\tilde{Z_{\text{In}}}\cdot \tilde{W_{In\;H1}}\cdot \tilde{W_{H1\;H2}}\cdot \tilde{W_{H2\;\mathrm{Out}}}$$

where $\tilde{Z_{\text{In}}}$ means the vector of all the neurons in input layer, $\tilde{W_{In\;H1}}$ means the connection vector between Input layer and H1 layer, $\tilde{W_{H1\;H2}}$ means the connection vector between H1 layer and H2 layer, $\tilde{W_{H2\;\mathrm{Out}}}$ means the connection vector between H2 layer and Output layer. $\tilde{R_{\text{In}}}$ actually contains all the relevance messages from output neurons to input neuron because of the transmission between neurons, it will be used as the RS value between all the input neurons and output neurons.

### 2.4 Optimization of neural network

To easily calculate the RS corresponding to disease severity, the publicly available free toolbox Pyrenn (32) from Technische Universität München has been used to implement the neural network learning, one neuron was assigned in the output layer to construct a regression model (**Figure 1**). The targets is from 1 to 3, where 1 is for healthy person, 2 is for moderate patients, and 3 is for severe patients. When calculating the RS between immune cell counts and disease severity, the input vector is the cluster percentage of whole blood cells(red blood cells have been lysed) of patients (**S1 File**). When calculating the RS between the concentration of activation markers and disease severity, the input vector is the activation marker concentrations(all data were compressed with an arcsin*h* transformation with cofactor 5) of one cell type of patients (**S2 File**).

Because the disease severity is not an exact number, but a range of values, the accuracy was set as in **Figure S1** (in **S3 File**) to meet the actual situation of this regressive neural network. For instance, if Yt(the target)=1, when yt(the predictive value)-Yt<0.5, the forecast is deemed accurate, otherwise, it is considered inaccurate, the whole algorithm is shown in **Figure S1**. The K-fold cross validation was used in the optimization process of neural network, in order to make the validation set contain about 20% of the sample data, the value of k is set to 5. The avarage accuracy of 20 epoches(20~40 epoches) was used to evaluate the performance of the neural network. The dropout regularizaion has been used to prevent the neural network from overfitting. The detail of the optimization processes of the neural network are recorded in **S3 File**.

After the neural network learning, the value of RS will be achieved. For neural network with two hidden layers created by Pyrenn (30), the connection matrix $\tilde{{IW}^{1,1}}$ , $\tilde{{LW}^{2,1}}$ and $\tilde{{LW}^{3,2}}$ in Pyrenn are actually the connection weight of input layer to hidden layer 1, hidden layer 1 to hidden layer 2, and hidden layer 2 to output layer respectively. According to formula (7) described in methods, the RS is calculated by the following formula:


(8) $$RS=I\tilde{W^{1,1}}\cdot I\tilde{W^{2,1}}\cdot I\tilde{W^{3,2}}$$

In order to measure the contribution of each input neuron per se to the result, $\tilde{Z_{\text{In}}}$ is not included in this formula.

The source code for optimization of neural network and the RS calculation have been uploaded on the website: https://github.com/Zhu-0010/hello_world/branches.

### 2.5 Welch’s t-test

The Welch’s t-test was used to compare whether the difference between the two averages of RSs(between active marker expression of cells and severity of COVID-19) between group HC_W and group HC_ICU is significant. This test assummes that both groups of data are sampled from populations that follow a normal distribution, but it does not assume that those two populations have the same variance.

### 2.6 The pipeline for RS calculation

**Figure 2:**
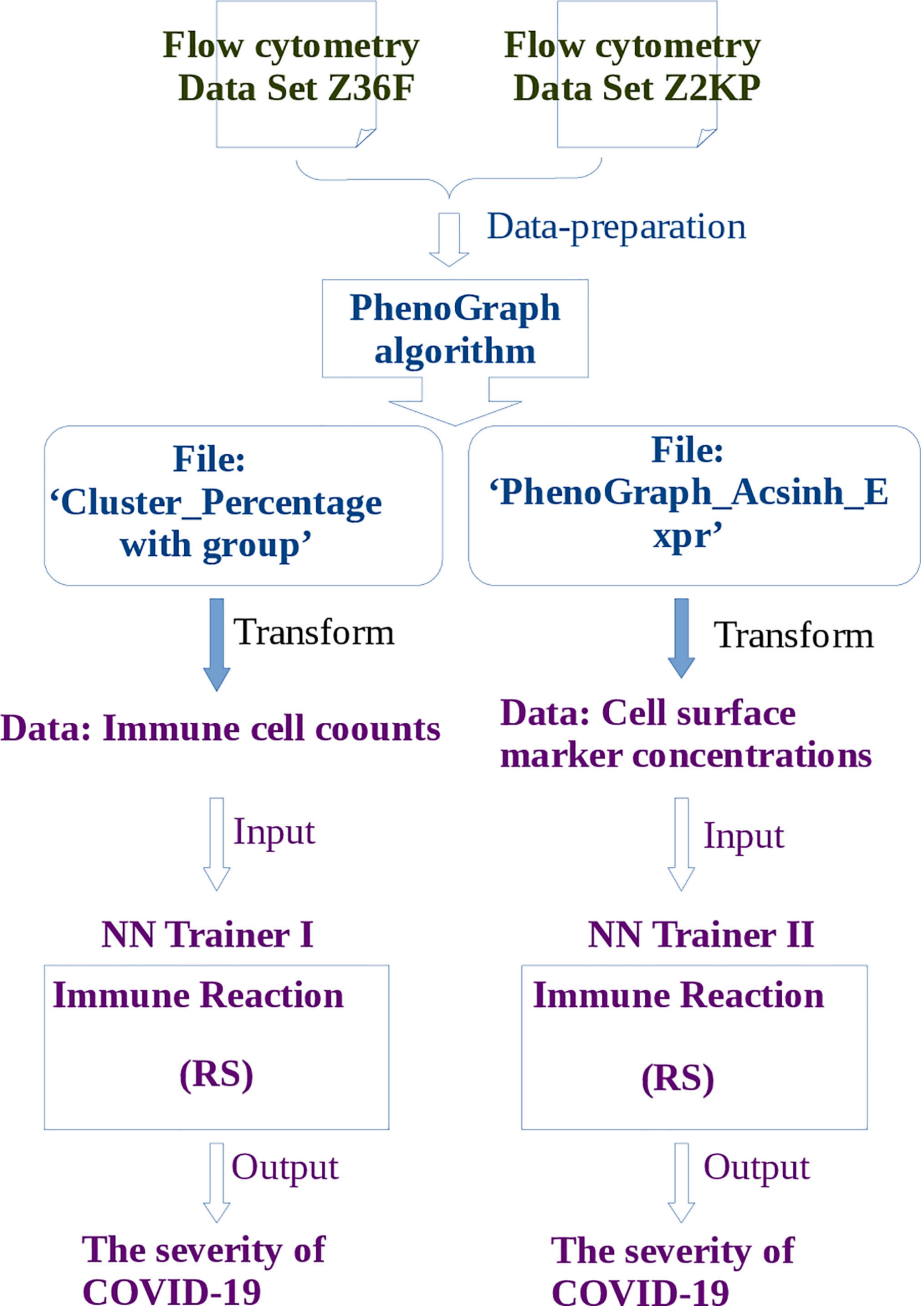
The pipeline of learning flow cytometry data by neural network. Where the operations described in blue words belong to steps of PhenoGraph Clustering, and the operations described in purple words belong to steps of neural network learning. The publicly available free toolbox Pyrenn (32) is used to implement the neural network learning.

The pipeline for RS calculation is carried out in the following order (**Figure 2**): firstly, the flow cytometry data of COVID-19 patients are prepared for PhenoGraph clustering, these preparations include filtering the margin events and gating the live single cells. Secondly, the PhenoGraph algorithm is used to robustly cluster these prepared data into phenotypically distinct subpopulations for each patient, and generates two files which will be used as the input data for neural network learning: the ‘Cluster_Percentage with group’ file and the ‘PhenoGraphX_Acsinh_Expr’ file, the former mainly contains the information of the immune cell(subpopulations) counts of each patient, the later mainly contains the activation marker concentrations on particular cells of each patient. Thirdly, these two files are transformed into the data format that is suitable for neural network learning. The main task is to determine the input and output neurons, where the data of cell counts or the activation marker concentrations are used as the input neurons, the disease severity are used as the output neurons. Fourthly, depending on the different kinds of input neurons, two neural networks are constructed. The RS between the input neurons and output neurons will be calculated according to the method described in subsection 4.2.

## 3 Results

### 3.1 Clustering by PhenoGraph algorithm

**Figure 3:**
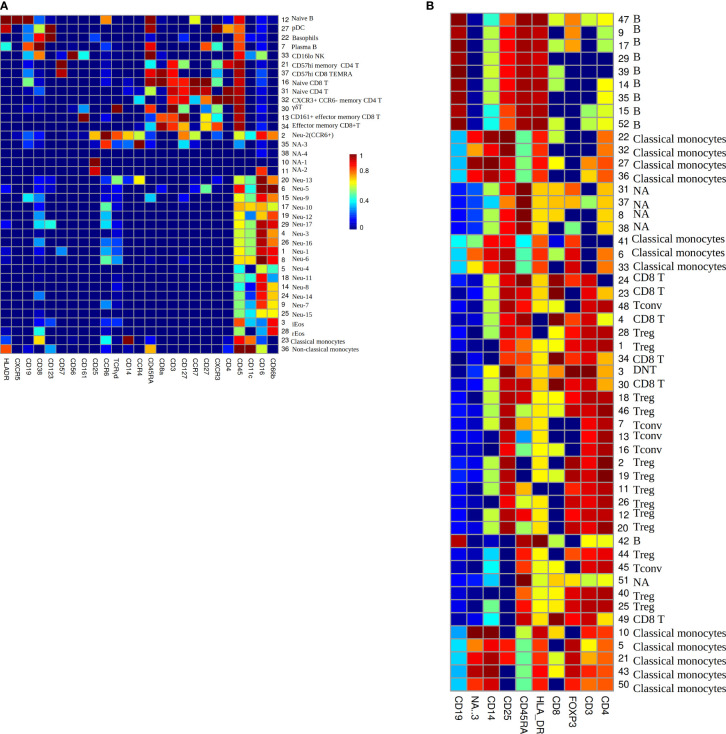
**(A)** The heatmap of median lineage marker expressed on automatically gated immune clusters of data set Z36F. **(B)** The heatmap of median lineage markers expressed on automatically gated immune clusters of data set Z2KP. The horizontal axis represent the types of markers. The vertical axis show the ID and manually gated cell type for each cluster, NA means there is no cell type matches this cluster by comparing the expression heatmap. The colors on the Mosaics from red to blue indicate the strongest to weakest marker intensity, the intensity of each kind of marker is normalized to 0-1.

To investigate the phenotype of immune cells in COVID-19 patients, the PhenoGraph algorithm (28) was used for robustly partitioning the two flow cytometry data set (**Table 1**) of COVID-19 patients into phenotypically distinct clusters. The algorithm identified 38 clusters for each patient in data set Z36F (**Figure 3A**), these clusters mainly included I B cells, plasmacytoid dendritic cELLs(pDC), basophils, plasma B cells, CD16 low NK cells, CD57 high memory CD4 T cells, CD57 high CD8 TEMInaive CD8 TIs, naive CD4 T cells, CXCR3+ CCR6- memory CD4 T cells, γδ T cells, CD161+ effector memory CD8 T cells, effector memory CD8 T cells, neutrophils, inducible eosinophils(iEos) (33), resident eosinophils(rEos), classical monocytes and non-classical monocytes. And the cell counts of these clusters for each patient have been gotten as well(**S1 File**), which will be used as the input data for deep learning of RS between immune cell counts and the severity of COVID-19, where the cell counts of immune cells will be used as the input neurons, and the severity of the patients will be used as the output neurons.

The PhenoGraph algorithm also identified 52 clusters for each patient in data set Z2KP (**Figure 3B**), the subtypes of these clusters included B cells, classical monocytes, CD8 T cells, conventional CD4 T cells (Tconv), regulatory CD4 T cells (Treg), double-negative T cells(DNT). And the activation marker concentrations of these clusters for each patient have been gotten as well (the activation markers’ information of cluster 1 are shown in **S2 File**), specifically, these activation markers are IL-17a, IL-2, GATA3, IFN-γ, IL-4, IL-10, PD_1, CD40L, RORγt, CTLA_4, CCR7, TNF-α and IL-6. These files will be used as the input data for deep learning of RS between activation marker concentrations and the severity of COVID-19, where the activation marker concentrations will be used as the input neurons, and the severity of patients will be used as the output neurons. These clusters’ cell types were defined by manual gating as described in Daniel et al. (34).

### 3.2 The RS between immune cell counts and the severity of COVID-19 at different stages

**Figure 4:**
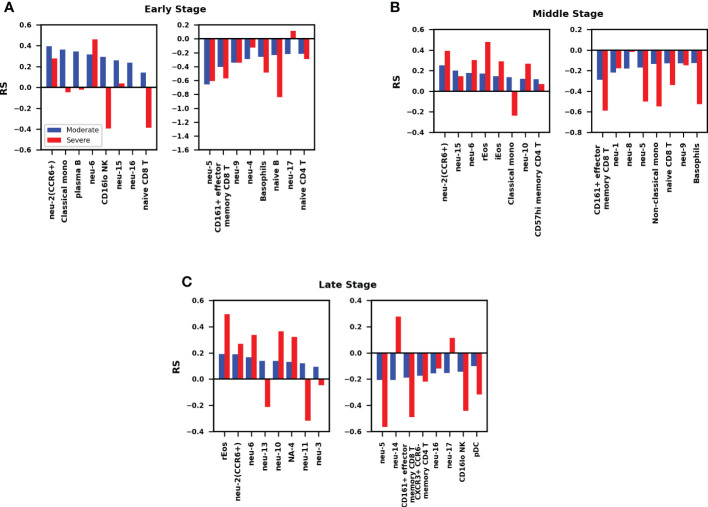
**(A)** Comparison of RS between moderate and severe patients at early stage. The RS represent the relevance score between cell counts and the severity of COVID-19. The mean accuracy in moderate patients is 69.5%, in severe patients is 61.2%. **(B)** Comparison of RS between moderate and severe patients at middle stage. The mean accuracy in moderate patients is 72.3%, in severe patients is 67.0%. **(C)** Comparison of RS between moderate and severe patients at late stage. The mean accuracy in moderate patients is 70.1%, in severe patients is 66.6.%.

Patients at different stages (in data set Z36F) and different severities are divided into six groups: health contral and moderate patients(HC_W)/health contral and severe patients(HC_ICU) at the early stage (day 1 since symptom onset), HC_W/HC_ICU at the middle stage (day 4), HC_W/HC_ICU at the late stage (day 7-12). The RS between immune cell counts of peripheral blood and severity of COVID-19 are calculated separately at these six stages. The hyperparameters of neural network are optimized the way in subsection 2.3, the network structure [n_in_, 5, 4, 1] with dropout rate = 0.1 was used for HC_W groups, and [n_in_, 2, 2, 1] with dropout rate = 0.2 was used for HC_ICU groups. The RS value is calculated through formula (8). The results are shown in **Figure 4**, a positive value of RS means that the cell counts have a positive correlation to the severity of COVID-19, the higher the RS value, the stronger the correlation. Relatively, a negative value of RS indicates that the cell counts have a negative correlation to the disease severity, the higher the absolute value, the stronger the negative correlation. What’s more, a RS value close to 0 is considered neutral, which means the cell counts has little influence on the severity of COVID-19 (20).

16 most correlated cell types (including 8 positive and 8 negative cell types) for each stage are recorded in descending order in **Figure 4**. We can get four aspects of information from this picture. Firstly, for moderate patients, the cell counts of immune cells in peripheral blood may increase or decrease during disease, it indicates that the decrease of immune cells in peripheral blood does not prevent the recovery of illness, it is more like a regular pathological process of COVID-19. Secondly, with the development of disease, the cell types work with positive/negative RS show the tendency of inheritance and development. For instance, in the early stage, CCR6+ neutrophils, classical monocytes, plasma B, CD16 lowIcells, naive CD8 T cells, neu-6, neu-15, and neu-16 show positive RS, in the middle stage, CCR6+ neutrophils, classical monocytes, neu-6, nue-15 still have positive RS with the severity of illness, while rEos, iEos, neu-10, and CD57 high memory CD4 T cells become outstanding, in the late stage, rEos, CCR6+ neutrophils, neu-6, neu-10 continue the positive relationship, the other neutrophils begin to work. Thirdly, as the disease progresses, immune cells with a positive RS value and those with a negative value rarely appear in the opposite camp, that suggests the orderliness of the immune response in moderate patients.

Fourthly and importantly, the most significant difference of RS between moderate and severe patients occurred at the early stage(**Figure 4A**). Cells that are positively correlated with the severity of COVID-19 in moderate patients but have no remarkable correlation in severe patients, they are classical monocytes, plasma B, and some subtypes of neutralphils, some even have negative correlation in severe patients, they are CI NK and naive CD8 T cells. The minor differences happened at the middle and late stage (**Figures 4B, C**), when the function of immune cells in moderate patients tended to be gentle, most of these cells in severe patients remained at a high level. In addition to the overall differences by stages, the classical monocyte, which increase significantly in moderate patients in the early and middle stage, but remained at a low level or even decreases in the severe patients.

### 3.3 The RS between activation marker concentrations and the severity of COVID-19

**Figure 5:**
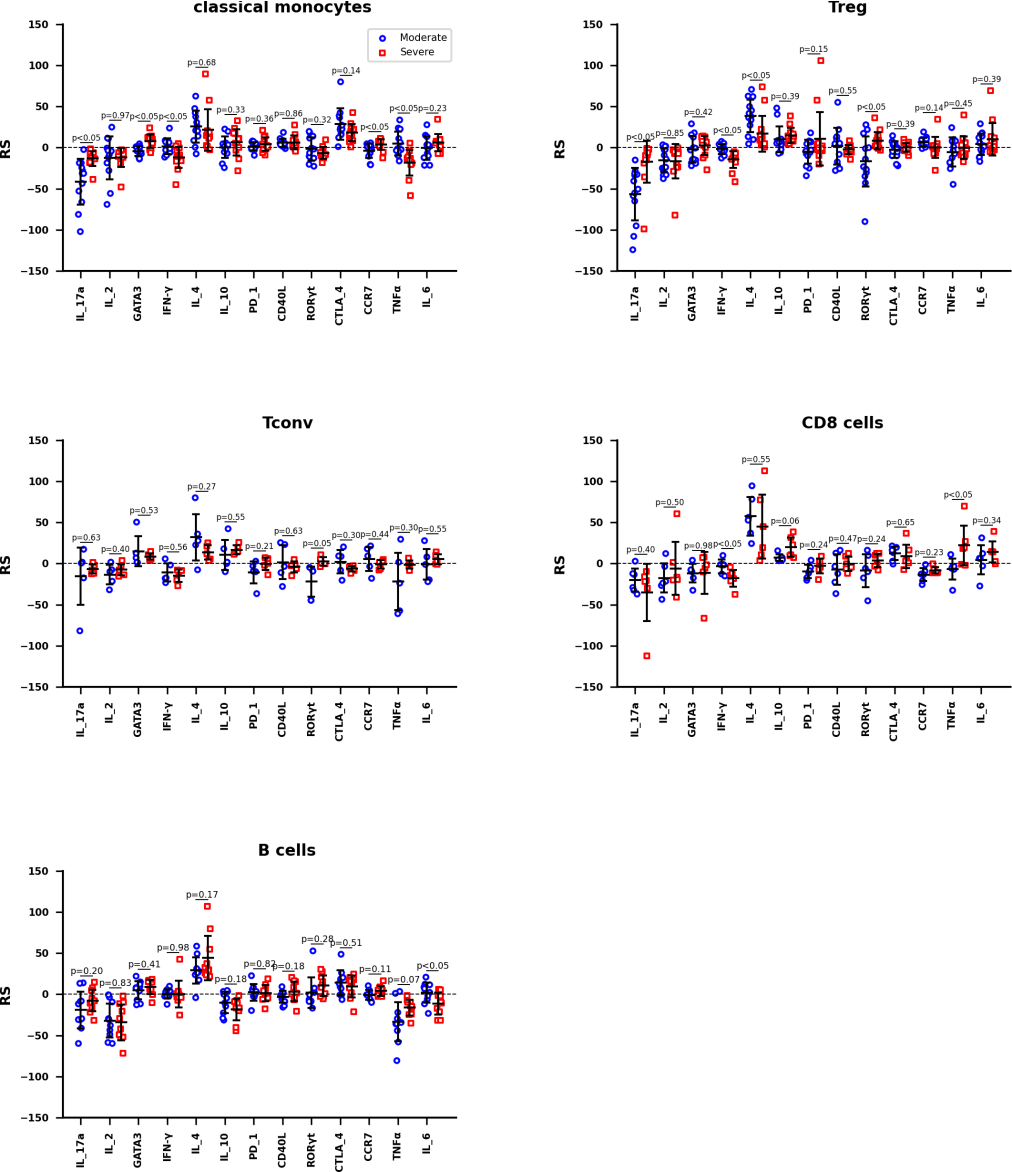
The RS between activation marker concentrations and the severity of COVID-19 of Classical monocytes, Treg, Tconv, CD8 T cells, and B cells, respectively. The mean accuracy of these neutral networks is 73.4% for moderate patients and 81.5% for severe patients. Differences are tested using Welch’s t-test.

The RS between activation marker concentrations and the severity of COVID-19 are calculated by training data set Z2KP, where the activation markers are IL-17a, IL-2, GATA3, IFN-γ, IL-4, IL-10, PD_1, CD40L, RORγt, CTLA_4, CCR7, TNF-α and IL-6, respectively. The hyperparameters of neural network are optimized the way in subsection 2.3, the network structure [n_in_, 1, 11, 1] was used for both HC_W and HC_ICU groups. The RS value is calculated through formula (8) as well. In **Figure 5**, each data point represent the RS of a subtype of one kind of immune cells, these immune cells are classical monocyte, Treg, Tconv, CD8 T and B cells. And the Welch’s t-test was used to determine whether the difference between the two averages of HC_W group and HC_ICU group is significant.

The information reflected in **Figure 5** can be interpreted in three ways. We hypothesize that the immune response in moderate patients is normal pathological and that in severe patients is abnormal pathological. Therefore, firstly, we focused on the expression of activation marker on immune cells in moderate patients. For classical monocytes, IL_4 and CTLA_4 are markedly positively correlated with the development of disease, while IL_17a shows a negative correlation. For Treg, IL_4 is positively correlated with this disease, IL_17a is negatively correlated. For both Tconv and CD8 T cells, IL_4 still shows a remarkable positive correlation. For B cells, IL_4 continues its significant positive correlation with disease, while IF_2 and TNFα show negative correlation with this disease. It is obvious that IL_4 is up-regulated in all cells, IL_17a, IF_2 and TNFα are down-regulated depending on cell types.

Secondly, we focus on the expression of activation markers that are significantly associated with moderate patients but not severe patients. In severe patients, IL_17a is not down-regulated on classical monocytes; none of the IL_17a and IL_4 is down or up regulated on Treg; and there are no obvious expression difference on Tconv, CD8 T, and B cells. These phenomenon suggest that the not down-regulation of IL_17a on classical monocytes and Treg are more associated with the occurrence of severe disease.

Thirdly, we focus on the expression of activation markers that are significantly associated with severe patients but not moderate patients. In severe patients, IFN-γ and TNFα are significantly down-regulated on classical monocytes; IFN-γ is down-regulated on Treg; IFN-γ is down-regulated but TNFα is up-regulated on CD8 T cells. These phenomenon imply that the down-regulation of IFN-γ on classical monocytes, Treg, and CD8 T cells are highly correlated with the occurrence of severe disease.

### 3.4 Dynamics of immune response in COVID-19 patients

**Figure 6:**
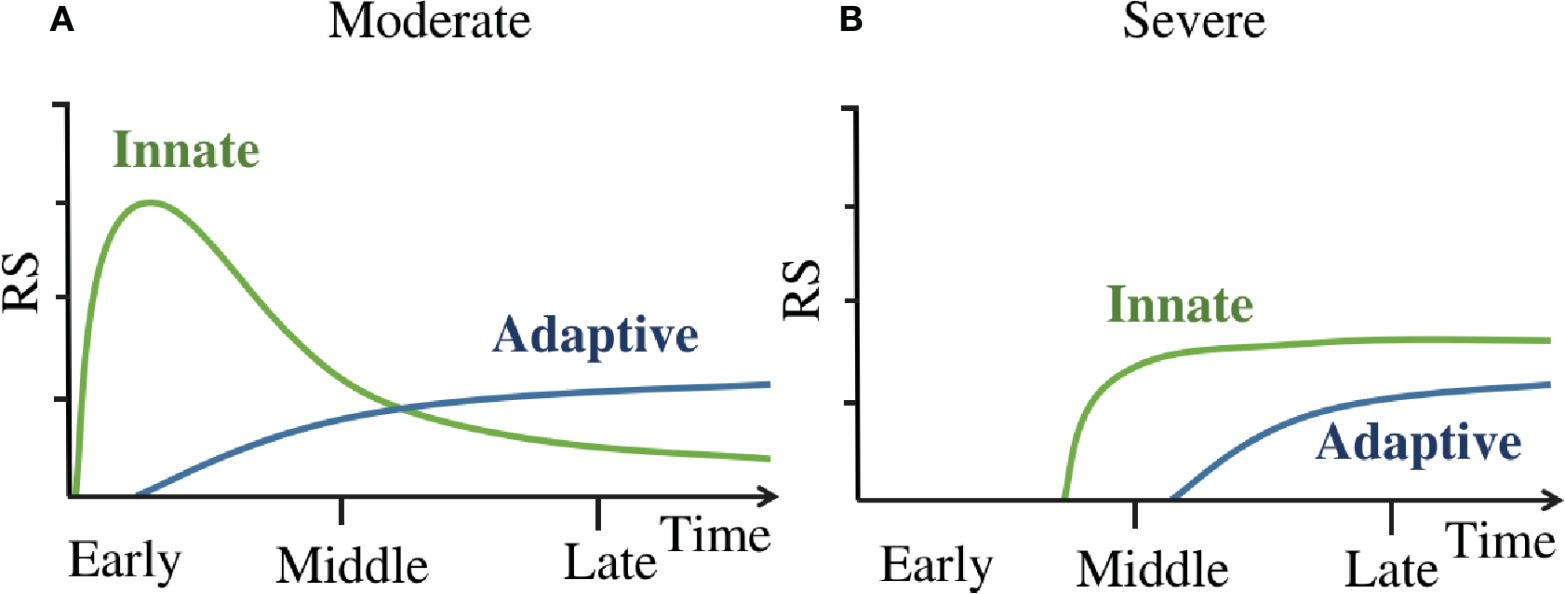
**(A)** The dynamics of immune response in moderate patients. **(B)** The dynamics of immune response in severe patients. ‘Innate’ line refers to the kinetics of innate immune cell counts detectable in peripheral blood and the activation marker concentrations of these cells. ‘Adaptive’ line refer to the kinetics of adaptive immune cell counts detectable in peripheral blood and the activation marker concentrations of these cells.

The dynamics of immune response in COVID-19 patients are summarized as a concise mode in **Figure 6**. This model suggests that in moderate patients (**Figure 6A**) the innate immune responses are rapidly activated on a large scale at the early stage, they occurred within one day since symptom onset. Then the adaptive immune responses are primed by the innate immune responses (35), it takes about several days (36) to generate enough virus-specific immune cells. Subsequently, the innate immune responses are down regulated after an early peak, and then these responses slowly decline and continue into late stage of disease. While in severe patients(**Figure 6B**), the innate immune responses are delayed till the middle stage of disease, and this leads to the delayed priming of adaptive immune responses as well. Once activated, the innate immunity remains highly activity(compared to moderate patients at the same time) till the late stage of disease. Then the adaptive responses are activated as well.

## 4 Discussion

This study calculated the RS between immune cell counts and the severity of COVID-19, and the RS between activation marker(transcription factors and cytokines) concentrations of immune cells and the severity of COVID-19. By comparing the RS of immune cells counts to the severity of COVID-19 between moderate and severe patients at different stages, we found that the innate immune responses in severe patients are delayed till the middle stage of disease, and this leads to the delayed priming of adaptive immune responses as well. The dynamics of immune response of COVID-19 found in our work are in consistent with the model reviewed by Alessandro Sette et al. (35) This model suggested that the immune evasion of SARS-CoV-2 makes it evade the triggering of early innate immune responses in severe patients, and this delay in innate immune responses is correlate with the severity of illness by failing to prime an adaptive immune response, what’s worse, the innate immune system (especially the neutrophils) tries to fill the vacuum left by absence of adaptive immune responses in the late stage, this finally result in excessive lung immunopathology. While our result show that not only the proliferation of a large number of neutrophils in the peripheral blood, but also the reduction of classical monocytes are significantly correlated with severe illness of COVID-19, this is consistent with other works (37, 38), both the apoptosis of classical monocytes in the circulatory system and migration to tissues are thought to influence the reduction of cell counts in the peripheral blood.

In addition, Daniel K. Beyer et al. (39) have reviewed that the delayed type I and type III IFN responses are associated with risk of severe COVID-19, and SARS-CoV-2 is thought to be effective at evading the triggering of early innate immune responses. And this delayed innate immune response subsequently failed to prime an adaptive immune response, the study of Carolina Lucas et al. (40) indicated that COVID-19 mortality did not correlated with the cross-sectional antiviral antibody levels per se but, rather, with the delayed kinetics of neutralizing antibody(NAb) production. What’s more, advanced age has been widely recognized as a significant factor associated with severe disease, multi-omics profiling (41) suggests that age may delay or impair antiviral cellular immune responses and delay efficient return to immune homeostasis. The above data linked the general evidence for the pathogenesis of SARS-CoV-2, for instance, the target: type I and type III IFN; the mechanism: delaying the innate immune responses; the immunological characteristics: the delayed innate immune cell counts and the delayed kinetics of NAb production; and the clinical characteristics: advanced age.

Apart from the dynamics of immune response clarified by our study, there are also some details worth noting. Firstly, the negative value of RS in **Figure 4** means that the corresponding cell counts have a negative correlation with the severity of COVID-19, these correlations corresponding to lymphopenia. An apoptosis and migration scoring system studied by Ji-Yuan Zhang et al. (42) suggested that cell death and lymphocyte migration (into infected site or adhesion to inflamed vascular endothelium) may be both associate with lymphopenia. So in our work, this negative value of RS mainly represent the decrease of the corresponding cells in the peripheral blood, the specific direction(apoptosis or migration) of these cells is till unknown.

Secondly, the lymphopenia is not only occurred in severe patients, but also occurred in moderate patients (**Figure 4**). In addition, lymphopenia is also occurred in patients with respiratory viral infections, such as the A/H3N2 virus, the human rhinovirus (HRV) and respiratory syncytial virus (RSV) (43). The above phenomenon imply that the lymphopenia is not the cause of severe illness.

Thirdly, it can be seen from **Figure 4** that the neutrophil subsets show strong correlation at different disease stages, but have different modes of action, including positive or negative correlations. This is not surprising because different neutrophil subsets act in heterogeneous manners have already been reviewed (44), the reactive oxygen species (ROS) and neutrophil extracellular traps (NETs) produced by neutrophils are thought to contribute to cell death (45), and neutrophils have been characterized in the lungs and tracheal aspirates of COVID-19 patients (8).

Deep learning of disease characteristics are often limited by the sample size and the complexity of patient’s own physical condition, its accuracy is usually not high (46–48). Our research is affected by the same factors, generally speaking, there are two main reasons that limited the accuracy of this study, one is the regression neural network model, the other is the sample size. Generally, the accuracy of classifier is higher than regression neural network, but one neuron was set in the output layer for facilitating the RS calculation, this made regressive neural network an appropriate choice for our work, and led to a partial sacrifice of accuracy. This work totally analyzed 145 cases, although it is sufficient for clinical analysis of diseases, it is indeed a small sample for neural network. Flow cytometry data set from the Flow Repository website often contains only dozens to hundreds of cases. Meanwhile, different cell staining strategies limit the possibility of merging these data sets to study homogeneity, they greatly limit the size of sample collection. Therefore, further validation of our results is warranted when additional data are released, as well as immunological data on infected sites, to help accurately interpreting the biological significant of negative RS.

## Data availability statement

The original contributions presented in the study are included in the article/**Supplementary Material**. Further inquiries can be directed to the corresponding authors.

## Ethics statement

Ethical review and approval was not required for the study on human participants in accordance with the local legislation and institutional requirements. Written informed consent for participation was not required for this study in accordance with the national legislation and the institutional requirements.

## Author contributions

TC assist in the analysis of immunological parameters. XM, YF and HS help validate machine learning results. D-QW assist in reviewing this article. GJ provide experimental guidance. All authors contributed to the article and approved the submitted version.

## Supplementary material

S1 File Cluster Percentage. The cluster counts(%) for each patient robustly clustered by PhenoGraph algorithm.

S2 File PhenoGraph1_Acsinh_Expr. The activation marker concentrations(simpleAsinh: asinh(x/5)) on PhenoGraph1(Cluster1) of each patient robustly clustered by PhenoGraph algorithm.

S3 File Optimization of the neutral network.
