# Supplementary material for: Machine learning of flow cytometry data reveals the delayed innate immune responses correlate with the severity of COVID-19
Source: Front Immunol. 2023 Jan 26;14:974343. doi: 10.3389/fimmu.2023.974343 (PMC9951775; doi:10.3389/fimmu.2023.974343)
Supplement: S3 File — Optimization of the neutral network. [file DataSheet_3.pdf]

S3 : Optimization process of the neural network

The accuracy algorithm was show in Fig S1, if the predicted value falls within the effective range of the target, this prediction was judged valid, otherwise invalid.

K-fold cross validation has been used in this neural network, where k=5, the acuracy of one epoch was the mean value of this 5 fold validations. We trained 500 epoches and recorded the performance of this model for each epoch(Fig S2), it can be seen from the Fig S2 that the validation accuracy remained stable after 20 epoches. Therefore, the mean validation accuracy from 20~40 epoches will be used as the evaluation standard for the following optimization processes.

For the neural network, it assumes the premise that the elements are independent for each other. But in the real world, many elements are related, such as a text, language and so on. The essence of RNN is that it has memory just like human beings, and it is especially suitable for learning data with correlation among elements. We use RNN to learn COVID-19 data and compare it with NN to judge whether there is correlation between cell types or cell markers. The accuracy of neural network for each data set was compared with that of the recurrent neural network(RNN) with one hidden layer, the results are shown in Fig S3, it shows that the neural network do better than the recurrent neural network with dInput = [0], dInter = [1], dOut = [1]. The experimental results showed that the prediction result of RNN is not ideal, which may be caused by the increasing complexity of parameters by RNN algorithm and further deepened the overfitting, it also indicate that the correlation between elements is not strong. As the number of hidden neurons increases, the validation accuracy decreases, that may be due to the complex neural network aggravating the overfitting.

Therefore, we will use neural network in the following experiments, then optimize the neural network structure, which include the number of hidden layers and the neuron numbers of each layer. As is shown in Table S1-S3, the neural network with two hidden layers do better than that with one hidden layer. Furthermore, in Table S1 of HC_W groups, the neural network with 5 neurons in hidden layer 1 and 4 neurons in hidden layer 2 performs better than the others. Therefore, the

regression neural network with the structure [n_{in}, 5, 4, 1] will be used for HC_W groups, [n_{in}, 2, 2, 1] will be used for the HC_ICU groups of data set Z36F. For data set Z2KP, the structure [n_{in}, 1, 11, 1] will be used.

After optimizing the structure of the neural network, we plan to use dropout regularizaion of the input layer to prevent the neural network from overfitting. The result are shown in Table S4, it shows that the best dropout rate for HC_W groups of data set Z36F is 0.1, the best dropout rate for HC_ICU groups of data set Z36F is 0.2. The best dropout rate for data set Z2KP is 0.

```
accuracy_cnt=0
for i in range(len(Yt)):
  if Yt[i] == min(1,2,3):
    if yt[i]-Yt[i] < 0.5:
      accuracy_cnt += 1
  elif Yt[i] == max(1,2,3):
    if yt[i]-Yt[i] > -0.5:
      accuracy_cnt += 1
  else:
    if abs(yt[i]-Yt[i]) <= 0.5:
      accuracy_cnt += 1
Accuracy=float(accuracy_cnt)/len(Yt)
```

Fig S1. The algorithm of accuracy calculation, where ‘accuracy_cnt’ means the number of successful predictions times, ‘Yt’ means the target value, ‘yt’ means the predictive value.

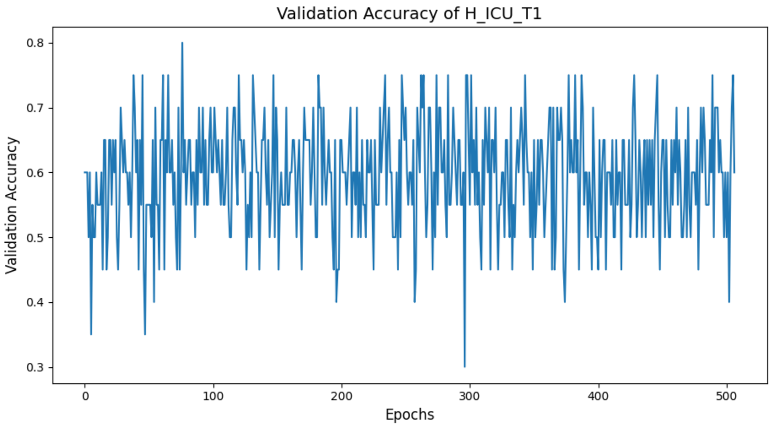

Fig S2. The validation accuracy of each epoch of HC_ICU_T1 group.

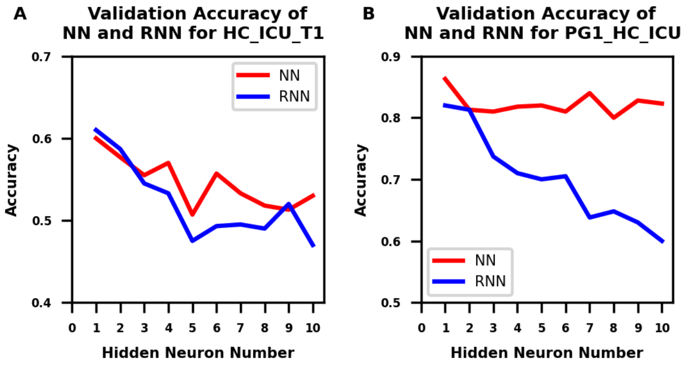

Fig S3. The validation accuracy of neural network(NN) and the recurrent neural network(RNN) for HC_ICU_T1 group of data set Z36F(A) and PG1_HC_ICU of data set Z2KP(B), where the hyperparameter for RNN is dInput=[0], dInter=[1], dOut=[1].

Table S1. The validation accuracy of group HC_W_T4 with 2 hidden layers.

Validation Accuracy of HC_W_T4 group											
		Neurons numbers of the second hidden layer									
		1	2	3	**4**	5	6	7	8	9	10
Neurons numbers of the first hidden layer	1	0.66	0.668	0.694	0.656	0.684	0.69	0.652	0.674	0.648	0.664
	2	0.674	0.676	0.706	0.71	0.70	0.692	0.684	0.724	0.702	0.646
	3	0.652	0.734	0.706	0.71	0.722	0.716	0.706	0.702	0.726	0.692
	4	0.7	0.722	0.708	0.736	0.696	0.762	0.73	0.726	0.72	0.744
	**5**	0.706	0.732	0.716	**0.774**	0.764	0.758	0.706	0.748	0.72	0.714
	6	0.75	0.762	0.702	0.748	0.748	0.742	0.716	0.72	0.742	0.76
	7	0.708	0.728	0.762	0.758	0.74	0.716	0.73	0.73	0.726	0.696
	8	0.706	0.732	0.706	0.76	0.754	0.724	0.716	0.732	0.708	0.714
	9	0.680	0.752	0.736	0.738	0.742	0.742	0.714	0.744	0.724	0.722
	10	0.678	0.738	0.73	0.742	0.732	0.72	0.712	0.728	0.744	0.74

Table S2. The validation accuracy of group HC_ICU_T4 with 2 hidden layers.

Accuracy of HC_ICU_T4 group											
		Neurons numbers of the second hidden layer									
		1	**2**	3	4	5	6	7	8	9	10
Neurons numbers of the first hidden layer	1	0.685	0.673	0.680	0.680	0.667	0.687	0.665	0.665	0.685	0.673
	**2**	0.687	**0.693**	0.658	0.682	0.700	0.673	0.645	0.695	0.630	0.653
	3	0.660	0.673	0.625	0.665	0.665	0.682	0.675	0.695	0.655	0.660
	4	0.702	0.660	0.643	0.630	0.638	0.628	0.665	0.650	0.595	0.640
	5	0.660	0.687	0.600	0.647	0.625	0.635	0.660	0.605	0.602	0.595
	6	0.670	0.660	0.665	0.665	0.600	0.642	0.625	0.63	0.645	0.63
	7	0.650	0.643	0.645	0.635	0.623	0.625	0.615	0.585	0.592	0.58
	8	0.640	0.668	0.630	0.630	0.620	0.585	0.605	0.625	0.573	0.53
	9	0.640	0.667	0.625	0.655	0.630	0.6	0.612	0.568	0.558	0.617
	10	0.653	0.668	0.645	0.605	0.585	0.602	0.602	0.57	0.58	0.558

Table S3. The validation accuracy of PG22_HC_W group of dataset Z2KP with 2 hidden layers.

Accuracy of PG22_HC_ICU for dataset Z2KP													
		Neurons numbers of the second hidden layer											
		1	2	3	4	5	6	7	8	9	10	**11**	12
Neurons numbers of the first hidden layer	**1**	0.803	0.803	0.795	0.775	0.813	0.785	0.793	0.813	0.788	0.828	**0.83**	0.795
	2	0.753	0.753	0.77	0.715	0.745	0.698	0.765	0.718	0.708	0.705	0.713	0.708
	3	0.74	0.715	0.698	0.713	0.685	0.705	0.665	0.663	0.665	0.66	0.653	0.653
	4	0.66	0.702	0.68	0.675	0.663	0.665	0.678	0.683	0.683	0.635	0.668	0.642
	5	0.67	0.673	0.713	0.678	0.673	0.665	0.615	0.64	0.653	0.628	0.593	0.653
	6	0.682	0.675	0.668	0.655	0.655	0.64	0.625	0.653	0.63	0.63	0.625	0.655
	7	0.66	0.695	0.678	0.653	0.663	0.62	0.67	0.597	0.58	0.658	0.583	0.628
	8	0.665	0.7	0.665	0.633	0.64	0.64	0.655	0.633	0.625	0.615	0.613	0.648
	9	0.625	0.658	0.675	0.685	0.663	0.61	0.695	0.638	0.605	0.633	0.615	0.608
	10	0.633	0.688	0.678	0.67	0.64	0.618	0.625	0.655	0.643	0.617	0.643	0.624

Table S4: The validation accuracy with the dropout rate from 0~0.5 for HC_W_T4, HC_ICU_T4, and PG22_HC_ICU.

Validation Accuracy	
dropout rate	HC_W_T4
0	0.740
**0.1**	**0.754**
0.2	0.744
0.3	0.718
0.4	0.726
0.5	0.680

Validation Accuracy	
dropout rate	HC_ICU_T4
0	0.670
0.1	0.655
**0.2**	**0.702**
0.3	0.683
0.4	0.685
0.5	0.685

Validation Accuracy	
dropout rate	PG22_HC_ICU
**0**	**0.795**
0.1	0.722
0.2	0.653
0.3	0.692
0.4	0.645
0.5	0.595
